# Differential Analysis of Psychopathological Impact of Cyberbullying in University Students

**DOI:** 10.3389/fpsyg.2019.01620

**Published:** 2019-07-17

**Authors:** Elena Felipe-Castaño, Benito León-del-Barco, Ma Isabel Polo-del-Río, Santiago Mendo-Lázaro, Teresa Gómez-Carroza, Fernando Fajardo-Bullón

**Affiliations:** Departamento de Psicología y Antropología, Facultad de Formación del Profesorado, University of Extremadura, Cáceres, Spain

**Keywords:** cyberbullying, cyberbullies, cybervictims, college students, psychopathological symptoms

## Abstract

The new technologies (NT) and Internet are now a part of our lives and they are even changing the way in which we relate to each other, in both a positive and a negative sense, especially among young people. One of the negative aspects is their use to harass others, a phenomenon known as Cyberbullying. The aim of this study was to describe the frequency of cyberbullying, the characteristics of victims and aggressors in a sample of university students, and to analyze the relationships between the use of Internet and the presence of psychopathological symptomatology, as well as the differences in the psychopathological dimensions in relation to the intensity of the cyberbullying, cyberaggression and gender. The participants were 1108 university students selected using a randomized cluster sample. The results demonstrate the presence of cyberbullying in our participants. No differences were found with respect to gender in the frequency of being a victim; but differences were found in this respect in the case of the aggressors, as well as there being different symptomatology profiles in males and females and according to the intensity of the aggression. The results are discussed in relation to the differences according to gender, as well as the need to carry out longitudinal studies and to design prevention and intervention programs for university campuses that are sensitive to the differences between males and females.

## Introduction

Cyberbullying is defined as intentional and recurrent aggression by a group or individual toward another individual, using electronic communication devices (Campbell, 2005; Smith et al., 2006).

Cyberbullying is considered to be a disguised form of verbal and written harassment (Mason, 2008), a characteristic it shares with traditional forms of harassment, but with some important differences, such as, for instance, that in cyberbullying there is nowhere you can be protected from it. In many cases, the harassment is public and may be observed indefinitely; the physical strength and size of the aggressor has no influence; the digital aggressor usually has good interpersonal relationships and cannot always be identified (Ybarra and Mitchell, 2004; Li, 2008; Ortega et al., 2008; Slonje and Smith, 2008; Heirman and Walrave, 2009).

Cyberbullying can be classified according to the means by which it is produced (Smith et al., 2006) and according to the type of harassment performed (Willard, 2006). Any kind of technological device and Internet can be used for this type of harassment: e-mail, mobile, social networks, instant messaging, and web pages. It must also be taken into account that, in cyberspace, the forms of harassment change and are reinvented as technological tools and resources develop.

The first studies on cyberbullying were published in the United States at the end of the last century and focused on the adolescent population (Finkelhor et al., 2000), since it was considered that adolescence was a time of particular risk due to the increase in the ease of access to new technologies (Tokunaga, 2010). Nevertheless, we are now seeing that cyberbullying continues into the university years and even later (Misawa, 2011). Research carried out on university students establishes different frequency percentages, with percentages of those involved in situations of cyberbullying being between 20 and 60%, independently of the role assumed (Dilmac, 2009; MacDonald and Roberts-Pittman, 2010; Turan et al., 2011; Walker et al., 2011; Selkie et al., 2015). As for the differences with respect to gender there is no agreement concerning the victims: so, in the studies reviewed, it has been pointed out that there are no differences between male and female university students concerning cybervictimization (Balakrishnan, 2015); while others do find differences with respect to gender, pointing to a greater frequency of female victims and male aggressors in both adolescents (Li, 2006; Calvete et al., 2010) and university students (Dilmac, 2009; Schenk et al., 2013).

Interest in the study of the consequences of cyberbullying comes from research carried out into traditional school bullying. In general, people who suffer traumatic experiences or situations have different types of psychopathological symptomatology (van der Kolk, 2005; Briere et al., 2008). Traditional bullying at school sets off, in both victims and aggressors, clearly negative effects that make integration in the school environment and the normal development of learning more difficult, not to mention the negative consequences on physical and mental health, especially in the victims (Kumpulainen and Räsänen, 2000; Espelage and Holt, 2001; Ivarsson et al., 2005; Anderson and Hunter, 2012; Felipe-Castaño et al., 2013). In addition, there is a high probability that both the psychosocial maladjustment and the psychopathological symptomatology may last the rest of a person’s life (Kumpulainen et al., 2001). Cyberbullying in adolescents has the same negative effects on mental health as traditional bullying (Wolak and Finkelhor, 2006; Hinduja and Patchin, 2007, 2010; Tokunaga, 2010), and could have an even greater impact on the victim (Slonje and Smith, 2008; Smith et al., 2008), as there is a higher correlation with suicidal behavior patterns and depression than traditional bullying (Bonanno and Hymel, 2013; van Geel et al., 2014).

University students involved as either victims or aggressors in situations of cyberbullying show an increase in depressive symptoms, alcohol consumption (Selkie et al., 2015) and a decrease in social skills (Kokkinos et al., 2014). Similar psychopathological profiles are to be found in aggressors and aggressors/victims, as well as an increase in anxiety and greater levels of distress in the students involved in comparison with those not involved (Schenk and Fremouw, 2012; Schenk et al., 2013).

Although more and more studies of cyberbullying in university students are becoming available, we believe it is necessary to continue acquiring more knowledge and better analyses of the characteristics and associated risk factors. Thus, the aim of this study was to describe the frequency of victims and aggressors involved in cyberbullying in a randomized sample of university students, and to analyze the relationship between the use of Internet and the Dimensions of the SA-45, as well as the differences in the psychopathological dimensions with respect to the intensity of the cyberbullying, cyberaggression and gender.

We will find differences between men and women regarding the experimentation of cyberbullying, so that women are more likely to be cyber-victims while men are cyber-aggressors. Then, the psychopathology associated with these profiles will be different for men and women. Men will present higher scores in dimensions related to externalizing symptoms such as hostility and psychoticism, while women will get higher scores in internalizing symptoms such as anxiety and depression.

## Materials and Methods

### Participants

There were 1108 university students participating in the study, of whom 655 were female (59%), between 18 and 41 years of age, with an average age of 20.95 (*SD* = 3.43), students from all 4 years of undergraduate studies, belonging to 40 different degree subjects from 14 different Faculties in the public universities. The number of participants was determined on the basis of the number of students registered in the previous year, considering a sample error of 3% and a confidence interval of 95%. A randomized cluster sampling was carried out using the Center or Faculty as the unit, while in each degree subject two class groups of students were randomly selected.

### Instruments

#### Sociodemographic Data and Information About the Use of Internet

Questions referred to gender, age, year of studies, and Center or Faculty, whether they owned a computer and cell phone; How much time was spent dedicated to Internet in hours (daily and weekly), use and profiles in social networks.

#### Scale of Victimization Through Internet (CYB-VIC, Buelga et al., 2012)

This is made up of ten items with a Likert type response scale with four intervals ranging from 1 (*never*) to 4 (*always*). This scale measures whether the participants have suffered from bullying according to the modalities, proposed by Willard (2006), of harassment, persecution, vilification, identity theft and violation, invasion of privacy and social exclusion. The value of internal consistency obtained using Cronbach’s α was 0.761.

#### Who Is the Aggressor?

The participants were asked who they considered the aggressor to be. The options to answer were: companions from the faculty, persons from outside the faculty, persons met through Internet, ex-friends or ex-partners, acquaintances, but unsure whether it was them or not, or strangers.

#### Duration

This refers to how long the aggression lasted. The options for answering were: 1 month or less, from 3 to 6 months, and a year or more.

#### Scale of Aggression Through Internet (CYB-AGRE, Buelga and Pons, 2012)

This scale is made up of ten items that evaluate aggressions committed over the previous year through Internet according to the modalities proposed by Willard (2006). It uses a Likert type scale with five response options ranging from 1 (*never*) to 5 (*many times*). The value of the reliability coefficient obtained through Cronbach’s α was 0.771.

#### Who Are Cyberaggressions Aimed at?

This question referred to who the victim was. The options for answering were: companions of the faculty, persons from outside the faculty, persons met through Internet, ex-friends or ex-partners, and strangers.

#### Duration

This question referred to the time the aggression was maintained. The options for answering were: a month or less, from 3 to 6 months, and a year or more.

#### SA-45 (Symptom Assessment-45 Questionnaire; Davison et al., 1997)

We used the adaptation of Sandín et al. (2008). This version maintains the same psychometric characteristics as the original version. SA-45 is a revised version of the original SCL-90 (*Symptom CheckList-90*; Derogatis et al., 1973) that evaluates the degree of psychological unease experienced by a subject through 45 symptoms that make up nine symptomatic dimensions: Depression, Hostility, Somatization, Obsession-compulsion, Interpersonal sensitivity, Anxiety, Phobic anxiety, Paranoid ideation and Psychoticism. The subject has to indicate how much each of the 45 symptoms has been present over the previous week, following a Likert type scale of five options for answering from 0 (*not at all*) to 4 (*a lot or extremely*). Internal consistency values were obtained from our participants, using the Cronbach’s α value, of 0.94 for the total score of the scale and in each symptomatology dimension: Somatization (α = 0.752), Obsession-compulsion (α = 0.741), Interpersonal sensitivity (α = 0.784), Depression (α = 0.783), Anxiety (α = 0.776), Hostility (α = 0.752), Phobic anxiety (α = 0.761), Paranoid ideation (α = 0.711) and Psychoticism (α = 0.701). This values were similar to original version.

### Procedure

Once the class groups had been selected, the teachers of the selected courses were contacted to gain permission to carry out the data gathering among the students during class hours. Once the permission had been obtained, we personally informed the collaborating teachers of the aims of the study and attended the classrooms at the agreed times. Once in the classroom, the researchers informed the participants of the aims of the research and the fact that their participation was voluntary, as well as the anonymous and confidential nature of the data collected. Finally, we asked for the participants’ informed consent in writing and asked them to respond sincerely to the questions.

Data collection was carried out over a 6 month period in the centers to which the students belonged, during class time and in the presence of the researchers. The time used to complete the questionnaires ranged from 30 to 40 min. No incident occurred during data collection that could affect the research.

### Data Analysis

The statistical package SPSS 20.0 for Windows was used to codify and analyze the data. To establish the different groups of intensity of cyberbullying, K-means clustering analyses were carried out. In order to analyze the differences of frequency between groups, contingency tables were created and we were using the Chi-square (χ^2^) test. We were used Pearson’s correlation to calculate the relationships between the variables. The analysis of the interaction between the intensity of the harassment and aggression and gender on the scores in the Dimensions of the SA-45 was carried out using a *MANOVA*, the value of the partial square (η^2^) was used as the size index of the effect. The calculation of the internal consistency of the scores in the Scales and Dimensions was performed using the value of Cronbach’s α.

## Results

### Use of Technological Devices and Internet

The percentage of participants who had a computer was the 98.5%, and 99% had a mobile phone, of whom 97% had access to Internet through the mobile phone. They dedicated between 0 and 17 h, with an average of 31.32 (*SD* = 23.62) hours a week and 4.47 h a day (*SD* = 3.37), to surfing the net. Virtually all participants (98%) had, at least, one profile in the social networks and of those 85% kept it up to date on a daily basis.

### Frequency of Cyber-Victimization and Cyber-Aggression, Duration and Persons Involved

The percentage of participants who had indicated having suffered at least one situation of cyber-bullying was 77.6%, while 51.2% stated they had committed at least one cyber-aggression over the previous year. As for the duration of the bullying, 19.5% (*n* = 218) was for 1 month or less, 1.7% (*n* = 19) between 3 and 9 months, and 0.9% (*n* = 10) a year or more. While for the aggressors, 15.5% (*n* = 172) was for a month or less, 1.3% (*n* = 14) between 3 and 9 months, and 0.9% (*n* = 10) a year or more.

In order to analyze the frequency of cases according to the intensity of the cyberbullying and gender, groups (low, moderate and severe), according to the score on the CYB-VIC and CYB-AGRE Scales (Table 1). To establish these groups we consider that the negative consequences of the victimization begin with a frequency of occurrence two to three times a month (Soldberg and Olweus, 2003). We found statistically significant differences in the frequency of aggressors, in the sense that there were more males in the moderate and severe groups of aggressors, while we also found more females in the low intensity aggression group (χ^2^(2) = 16.04; *p* < 0.000).

**Table 1:** CY-VIC and CY-AGRE group intensity by gender, frequency and percentage of participants, and descriptive statistics.

				Gender			
	
	Group	Total		Male		Female	
		*n* (%)	*M* (*SD*)	*n* (%)	*M* (*SD*)	*n* (%)	*M* (*SD*)
CY- VIC	L	616 (56)	10.88 (0.83)	267 (58.9)	10.78 (0.81)	349 (53.3)	10.97 (0.83)
	M	452 (41)	14.39 (1.44)	168 (37.1)	14.52 (1.53)	284 (43.4)	14.31 (1.38)
	S	40 (4)	21.55 (3.95)	18 (4)	22.39 (5.12)	22 (3.4)	20.86 (2.59)
CY-AGRE	L	709 (64)	10.23 (0.42)	265 (58.5)	10.22 (0.42)	444 (67.8)	10.24 (0.43)
	M	390 (35)	13.80 (2.24)	181 (40)	14.34 (2.60)	209 (31.9)	13.32 (1.75)
	S	9 (1)	33.11 (7.88)	7 (1.5)	34.29 (8.67)	2 (0.3)	29 (1.41)

*L: Low; M: Moderate; S: Severe.*

As for who carries out the harassment (Table 2), we find statistically significant differences in that women said they were harassed more frequently by ex-friends or ex-partners (χ^2^(1) = 5.24; *p* = 0.022), while men said they more frequently harassed faculty friends (χ^2^ (1) = 14.26; *p* < 0.000) and persons outside the faculty (χ^2^ (1) = 13.92; *p* < 0.000). It should be pointed out that 24.8% (*n* = 139) of the victims and 10.3% (*n* = 59) of the aggressors did not know who was harassing them or who they were harassing, or they were not sure.

**Table 2:** Frequency and percentage of participants: who harasses and who is harassed by gender – Chi-square (χ^2^) test.

	Cyber-victims			Cyber-bullies		
	Male	Female	χ^2^	Male	Female	χ^2^
	*n* (%)	*n* (%)		*n* (%)	*n* (%)	
Faculty friends	13 (2.9)	16 (2.4)	0.186	38 (8.4)	21 (3.2)	14.266**
Persons outside the faculty	39 (8.6)	52 (7.9)	0.151	78 (17.2)	63 (9.6)	13.928**
Ex-friends or ex-partners	43 (9.5)	92 (14)	5.248*	35 (7.7)	73 (11.1)	3.558
Known persons but unsure	33 (7.3)	59 (9)	1.066	11 (2.4)	17 (2.6)	2.322
Unknown persons	19 (4.2)	28 (4.3)	0.005	12 (2.5)	19 (2.8)	0.03
Total	147 (32.5)	247 (37.6)		174 (38.2)	193 (29.2)	

*^∗∗^*p* < 0.001; ^∗^*p* < 0.05.*

### Time Dedicated to Internet, CYB-VIC, CYBAGRESS and Score in the SA-45 Dimensions

The correlation coefficients were calculated between the scores in the SA-45 Dimensions and the hours dedicated to Internet per day by gender. We found direct and statistically significant correlations in males, although they were of low intensity, between the hours dedicated to surfing the net and all the SA-45 Dimensions, in the following order from greater to lesser intensity: Interpersonal sensitivity (*r* = 0.164; *p* < 0.001); Psychoticism (*r* = 0.141; *p* < 0.01); Phobic anxiety (*r* = 0.138; *p* < 0.01); Depression (*r* = 0.113; *p* < 0.01); Hostility (*r* = 0.109; *p* < 0.01); Paranoid Ideation (*r* = 0.102; *p* < 0.01) and Somatization, Anxiety and Obsession-compulsion (*r* = 0.100; *p* < 0.01). We found direct statistically significant correlations between the dimensions of the SA-45 and the score in CYB-VIC and CYB-AGRESS, both male and female (see Table 3).

**Table 3:** Correlation analysis.

	Daily		CYBVIC			CYBAGRESS		
	Male	Female	Male	Female	Total	Male	Female	Total
Depression	0.132^∗∗^	-0.057	0.271^∗∗^	0.270^∗∗^	0.270^∗∗^	0.183^∗∗^	0.213^∗∗^	0.166^∗∗^
Hostility	0.118^∗^	0.048	0.442^∗∗^	0.243^∗∗^	0.340^∗∗^	0.477^∗∗^	0.312^∗∗^	0.394^∗∗^
Interpersonal sensitivity	0.179^∗∗^	-0.009	0.366^∗∗^	0.238^∗∗^	0.292^∗∗^	0.308^∗∗^	0.199^∗∗^	0.212^∗∗^
Somatization	0.109^∗^	0.034	0.385^∗∗^	0.217^∗∗^	0.287^∗∗^	0.381^∗∗^	0.141^∗∗^	0.239^∗∗^
Anxiety	0.112^∗^	-0.064	0.460^∗∗^	0.233^∗∗^	0.335^∗∗^	0.380^∗∗^	0.157^∗∗^	0.244^∗∗^
Psychoticism	0.145^∗∗^	-0.060	0.480^∗∗^	0.236^∗∗^	0.356^∗∗^	0.429^∗∗^	0.221^∗∗^	0.337^∗∗^
Obsession-compulsion	0.111^∗^	0.011	0.271^∗∗^	0.234^∗∗^	0.249^∗∗^	0.177^∗∗^	0.180^∗∗^	0.144^∗∗^
Phobic anxiety	0.155^∗∗^	-0.043	0.378^∗∗^	0.237^∗∗^	0.298^∗∗^	0.431^∗∗^	0.190^∗∗^	0.288^∗∗^
Paranoid ideation	0.106^∗^	0.008	0.402^∗∗^	0.287^∗∗^	0.340^∗∗^	0.367^∗∗^	0.281^∗∗^	0.298^∗∗^

*SA-45 Dimensions and daily hours dedicated to surfing the Internet, and CYBVIC and CYBAGRESS by gender. ^∗∗^*p* < 0.001; ^∗^*p* < 0.05. CYB-VIC: Scale of victimization through Internet; CYB-AGRES.*

### Intensity of Cyberbullying and Cyberaggression by Gender

An analysis of the variance of the factors (*MANOVA*) was calculated in order to analyze the interaction between gender and intensity of the cyber-victim and cyber-aggressor on the scores in the SA-45 Dimensions and we found that the interaction was significant for the gender and cyber-victim groups (*Wilks*ʻ = 0.954; *F* = 2.89, *p* < 0.000, η^2^ = 0.023), in the sense that the observed differences in the SA-45 Dimensions between males and females are not the same in all the cyber-victim groups. To be precise, we found statistically significant differences in the dimensions of Hostility (*F* = 4.670; *p* = 0.010*;*η^2^ = 0.008), Anxiety (*F* = 6.769; *p* = 0.001; η^2^ = 0.012) and Psychoticism (*F* = 6.964; *p* = 0.001; η^2^ = 0.013), in the sense that the males obtained higher scores than the females in the dimensions of Anxiety of the severe victimization group and Psychoticism and Hostility in the severe and moderate victimization groups; while the females obtained higher scores in comparison to males in the dimension of Anxiety in the low and moderate victimization groups (Table 4). Males who described themselves as victims of cyberbullying obtained significantly higher scores than the females in Anxiety, Hostility and Psychoticism, which are conceived as clinical manifestations of anxiety and general signs of emotional tension and their psychosomatic manifestations, together with thoughts, feelings and behavior patterns characteristic in states of aggressiveness, anger and irritability as well as feelings of social alienation and isolation, with interpersonal difficulties.

**Table 4:** Mean and standard deviation: contrasts according to gender by cybervictim group.

*Gender*	*Male*			*Female*		
*Groups CIB-VIC*	*Low* (*n* = 264)	*Moderate* (*n* = 166)	*Severe* (*n* = 18)	*Low* (*n* = 348)	*Moderate* (*n* = 282)	*Severe* (*n* = 22)
	***M* (*SD*)**	***M* (*SD*)**	***M* (*SD*)**	***M* (*SD*)**	***M* (*SD*)**	***M* (*SD*)**

*Hostility*	1.90 (2.95)	3.47 (3.92)	8.50 (5.17)	2.03 (2.92)	3.16 (3.30)	5.41 (4.56)
*p*			0.003			0.003
*Anxiety*	2.10 (2.78)	3.57 (3.30)	8.28 (5.99)	3.11 (2.98)	4.47 (3.48)	5.41 (4.68)
*p*	0.000	0.004	0.005	0.000	0.004	0.005
*Psychoticism*	1.25 (1.96)	2.67 (2.85)	6.17 (5.02)	1.50 (2.07)	2.06 (2.36)	4.23 (3.49)
*p*		0.008	0.010		0.008	0.010

*CIB-VIC: Scale of victimization through Internet.*

## Discussion

The aim of this study was to describe the frequency and characteristics of cybervictims and cyberaggressors in a random sample of university students, and to analyze the relationships between the use of Internet and the SA-45 Dimensions, as well as the differences in the psychopathological Dimensions according to the intensity of the cyberbullying and cyberaggression and gender.

Practically all the participants had cell phones with access to Internet and used them daily, having at least one active profile in the social networks. Access to and use of Internet has become widespread since the appearance of smartphones, which allow rapid access similar to that of a PC.

The results show high percentages of cybervictims and cyberaggressors, but percentages which are lower than those found for university students in other countries (Dilmac, 2009; Turan et al., 2011; Selkie et al., 2015). The social and cultural characteristics of the different countries could explain these differences, as well as the use of different data gathering instruments based on different definitions of cyberbullying. We can conclude that cyberbullying takes place at all educational levels and is quickly becoming a social problem (Misawa, 2010) due, among other things, to the rapid development of the technological tools and the population’s quick and easy access.

As for differences regarding gender in the question of being victim or aggressor, our results coincide with those obtained by other researchers who find a greater frequency of male aggressors (Li, 2006; Smith et al., 2008; Dilmac, 2009; Calvete et al., 2010), but not with respect to the victims, since no differences were found between males and females, a result similar to that obtained by Balakrishnan (2015). It would be necessary to continue investigating, since much of the research work was carried out with samples of adolescents, while our results were obtained from university students, who are older and have different social characteristics.

As for the identity of the aggressor, a significant percentage of female participants were harassed by ex-friends or ex-partners. The break-up of interpersonal relationships, especially those of friendship or partners, could be another factor associated with the presence of cyberbullying, in addition to jealousy, envy and racial and sexual intolerance (Hoff and Mitchell, 2009). This fact connects directly with aspects related to gender violence in so far as the ICTs are used as another tool with which to continue the aggressions. We believe this aspect should be dealt with in particular through the need to control the behavior patterns of cyberbullying. It is also important to stress the fact that, in many cases, the aggressor cannot be identified, which means that the victim feels defenseless against the attacks and therefore the consequences can be even more devastating (Li, 2008; Heirman and Walrave, 2009).

Among males, we find a significant relation between the hours dedicated to surfing the net and the scores in the SA-45 Dimensions, but not so with females. In addition, we find different psychopathological profiles in men and women who describe themselves as victims. This result demonstrates that being a victim of cyberbullying is related with a different symptomatology in men and women. Male victims are characterized by greater anxiety, hostility and psychoticism. We should note the presence of anxiety, a psychopathological symptom in which women usually obtain higher scores than men (Derogatis, 2002; Sandín et al., 2008).

It still remains to be seen whether the presence of psychopathological symptoms are the cause or the consequence of the situations of harassment (Menesini et al., 2009). We also believe, however, that it would be necessary to investigate these results in greater depth through the design of longitudinal studies that would allow the behavioral and psychopathological antecedents to be established, as well as the consequences that these experiences may have on a person’s mental and physical health.

We consider it necessary to always analyze cyberbullying from the perspective of differences according to gender, as both the role played (where the aggressors are more often male) and the associated psychopathological correlations are different in men and women. Knowledge of the differential psychopathological profiles will be useful when it comes to designing prevention and intervention programs sensitive to the differences, as well as for the formulation of explanatory hypotheses concerning cyberbullying.

It can be concluded that generalized access to the ICTs by our adolescents and young adults is modifying the styles of interaction and interpersonal relationships. Face to face relationships are not the same as when one is not sure who the other person is or whether that person is really who they say they are. In our society, we depend to an ever greater extent on technological tools for studying, for social relationships and leisure; in fact, universities facilitate and recommend access to Internet and social networks as another tool in a person’s formation. Making a good use of these tools and being able to detect when there is inadequate behavior, as well as what to do in such circumstances, is essential for the protection of our students. It is, therefore, ever more necessary to implement prevention programs at all educational levels, especially in university, as this is a stage when access to Internet and social networks is especially facilitated, if not actually required, by the institution itself. We therefore agree with the proposal of Washington (2014) with respect to the idea that universities should develop informative programs and prevention and intervention procedures that will guarantee security, in the widest sense of the word, on campuses. In view of our results, we consider that psychotherapy interventions aimed at preventing cyberbullying in university context may best focus on specific pattern in males and females and specific levels of intensity of cyberbullying.

## Limitations

The study we have carried out has several limitations to the scope of the conclusions. This study was cross-sectional and therefore the relationship between variables cannot be examined theoretical, so the use of self-reporting in the information gathering, which may affect the honesty of the responses and the social desirability. Future work should have other instruments for data collection that can help to contrast the results, such as individual interviews or self-registration, and should use more sophisticated analysis to examine potential prediction the role of SA-45 Dimensions on use of Internet. Participants were, a priory, healthy and that the findings may not generalize to victims of cyberbullying diagnosed with post-traumatic stress disorder and belong to a group with very homogeneous educational and sociodemographic characteristics; it would therefore be necessary to complement this with other groups of non-university participants of the same age and with different social and demographic characteristics, as well as a different access and use profile. For these reasons, the results cannot be generalized to other population groups.

## Data Availability

All datasets generated for this study are included in the manuscript and/or the supplementary files.

## Ethics Statement

As for ethical norms, the study received approval from the Ethics Committee of the University of Extremadura. Prior to administering the questionnaire, following the ethical directives of the American Psychological Association (2009), the students gave their informed consent to participate in the research, guaranteeing the anonymity and confidentiality of the data and their exclusive use for research purposes.

## Author Contributions

EF-CKindly confirm whether the Author contribution section is fine. and BL-d-B analyzed and interpreted the data. EF-C,M-IP-d-R, and SM-L drafted the work. EF-C, BL-d-B, M-IP-d-R, TG-C, and FF-B conceived and designed the work.

## Conflict of Interest Statement

The authors declare that the research was conducted in the absence of any commercial or financial relationships that could be construed as a potential conflict of interest.
